# Tumor Lysis Syndrome Following Endoscopic Retrograde Cholangiopancreatography With Stent Placement in a Patient With Cholangiocarcinoma: A Case Report

**DOI:** 10.7759/cureus.79476

**Published:** 2025-02-22

**Authors:** Rim A Boutari, Mahmoud M Hallal, Fatmeh I Mallah

**Affiliations:** 1 Gastroenterology and Hepatology, Al Zahraa Hospital University Medical Center, Beirut, LBN; 2 Radiology, Faculty of Medicine, Lebanese University, Beirut, LBN

**Keywords:** biliary stent, case report, cholangiocarcinoma, ercp, oncological emergencies, tumor lysis syndrome

## Abstract

Tumor lysis syndrome (TLS) is an oncological emergency commonly associated with hematologic malignancies and aggressive chemotherapy. Its occurrence following endoscopic retrograde cholangiopancreatography (ERCP) with stent placement in patients with cholangiocarcinoma is exceedingly rare, with only a handful of cases reported in the literature. This report discusses a unique case of TLS occurring after ERCP in a patient with cholangiocarcinoma, highlighting the clinical challenges and the need for heightened awareness among clinicians. Herein, we present the case of a 58-year-old female patient with a diagnosis of cholangiocarcinoma who developed TLS following ERCP with stent placement. The patient initially underwent the procedure to relieve biliary obstruction, a common palliative intervention in cholangiocarcinoma cases. Within 24 hours post-procedure, the patient exhibited signs of acute renal failure, hyperkalemia, hyperphosphatemia, and hypocalcemia, consistent with TLS. Prompt recognition and aggressive management, including hydration, electrolyte correction, and renal support, were crucial in stabilizing the patient. Despite prompt recognition and aggressive management, including hydration, electrolyte correction, and renal support, the patient unfortunately passed away 24 hours later. This tragic outcome underscores the critical importance of early recognition and intervention, as well as the need for awareness of this rare but possible complication. This case highlights the rare but serious risk of TLS following ERCP in patients with cholangiocarcinoma. Clinicians should be aware of this potential complication, especially in patients with a high tumor burden or those undergoing palliative procedures. Early recognition and intervention are paramount in improving patient outcomes. Further studies are needed to better understand the mechanisms and predisposing factors for TLS in this context.

## Introduction

Tumor lysis syndrome (TLS) is a life-threatening oncologic emergency characterized by the rapid release of intracellular components into the bloodstream following the lysis of malignant cells. It is most commonly associated with hematologic malignancies, such as acute leukemias and high-grade lymphomas, particularly following the initiation of chemotherapy [1,2]. However, its occurrence in solid tumors, especially cholangiocarcinoma, remains exceedingly rare and often underrecognized. Cholangiocarcinoma, a malignant tumor arising from the bile ducts, is typically associated with a poor prognosis, especially when diagnosed at an advanced stage. The treatment options for cholangiocarcinoma often involve palliative measures aimed at relieving biliary obstruction, a common complication that can lead to jaundice, cholangitis, and liver failure [3]. Endoscopic retrograde cholangiopancreatography (ERCP) with stent placement is a widely used palliative procedure in such cases, and it helps to alleviate biliary obstruction and improve patient’s quality of life [4].

While ERCP is generally considered a safe and effective procedure, the development of TLS as a complication post-ERCP is extraordinarily rare. The pathophysiology of TLS in solid tumors, including cholangiocarcinoma, remains poorly understood. It is hypothesized that the rapid reduction of tumor mass, whether through chemotherapy, targeted therapy, or physical interventions such as stenting, may lead to the abrupt release of intracellular contents, precipitating the onset of TLS [5]. The Cairo-Bishop criteria, which combine laboratory and clinical findings, are crucial for the diagnosis of TLS and help in the early identification and management of this syndrome [6].

Understanding this phenomenon is crucial for early identification and prompt management, particularly in patients with significant tumor burden undergoing therapeutic interventions.

## Case presentation

A 58-year-old female, with a history of hypertension, presented with jaundice, abdominal pain, generalized fatigue, low-grade fever, and pruritus. Physical examination showed icteric sclera, cachexia, and right upper quadrant (RUQ) tenderness to palpation. Laboratory evaluation on admission revealed normal renal function and electrolytes at baseline; however, labs also showed hyperbilirubinemia, elevated alkaline phosphatase (Alk phos), gamma-glutamyl transferase (GGT) levels, and a high C-reactive protein (CRP) level (Table 1).

**Table 1:** Laboratory values on admission WBC: white blood cells, Alk phos: alkaline phosphatase, GGT: gamma-glutamyl transferase, CRP: C-reactive protein

Parameter	Value	Normal range
WBC	13560 cells/µL	4,000-11,000 cells/µL
Creatinine	0.7 mg/dL	0.6-1.2 mg/dL
Potassium	4.1 mmol/L	3.5-5.0 mmol/L
Phosphate	3.0 mg/dL	2.5-4.5 mg/dL
Uric Acid	4.5 mg/dL	3.5-7.2 mg/dL
Calcium	8.6 mg/dL	8.5-10.2 mg/dL
Direct bilirubin	9.2 mg/dL	< 0.3 mg/dL
Alk phos	394 U/L	45-115 U/L
GGT	276 U/L	9-48 U/L
CRP	116 mg/L	< 10 mg/L

**Figure 1:**
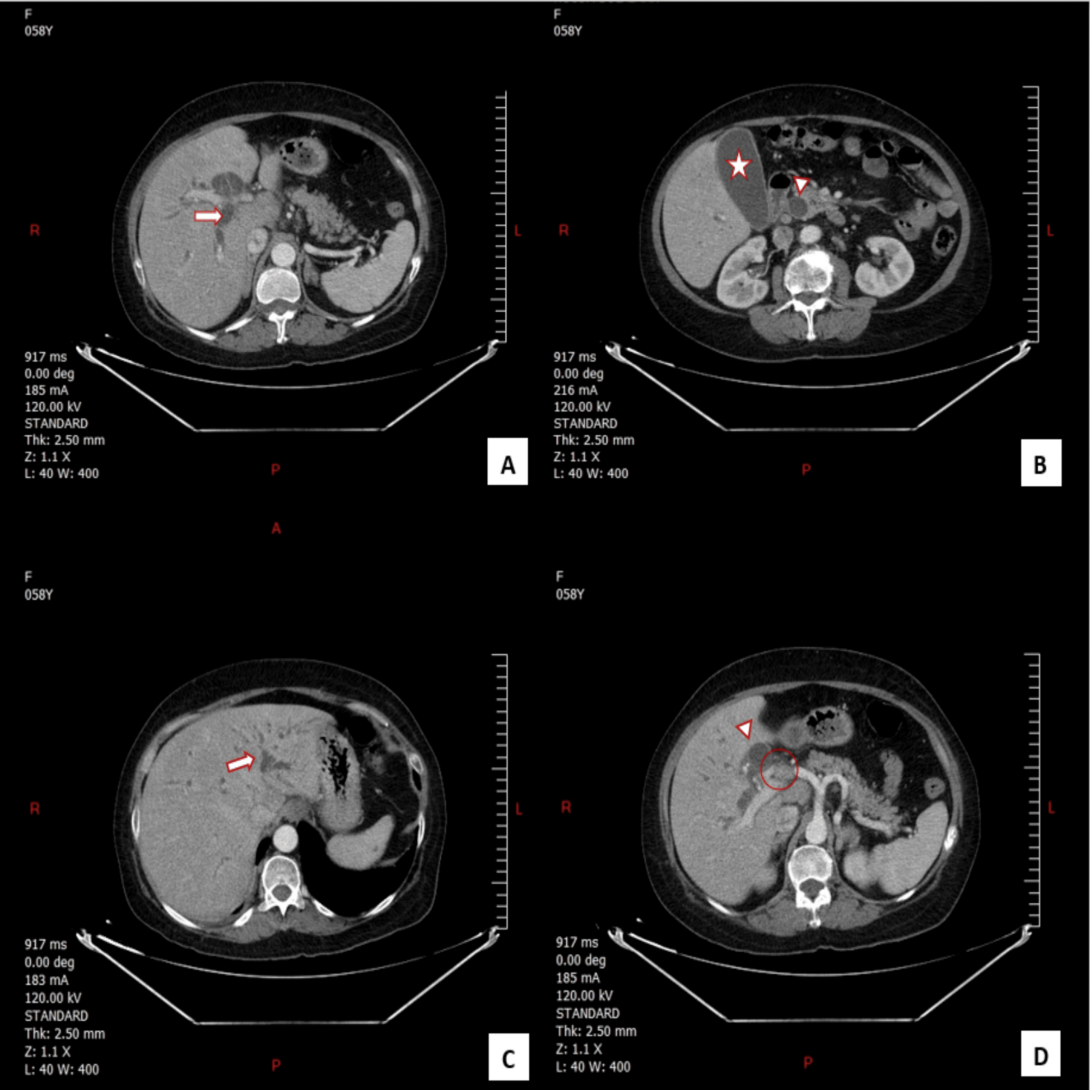
Contrast-enhanced CT scan of the abdomen revealing a mass consistent with obstructing cholangiocarcinoma Figures 1A, 1B, and 1C: CT imaging demonstrates significant dilation of the intra-hepatic (arrowhead) and extra-hepatic(arrow) bile ducts, with accompanying distension of the gallbladder (star), consistent with biliary obstruction secondary to cholangiocarcinoma. Figure 1D: An enlarged lymph node at the hepatic hilum is noted (circle), along with gallbladder distension, raising suspicion for regional metastasis and biliary obstruction in the context of cholangiocarcinoma.

The clinical picture suggested cholangitis. A contrast-enhanced CT scan of the abdomen revealed a mass consistent with obstructing cholangiocarcinoma (Figures 1A-1D).

After a multidisciplinary discussion, the decision was made to proceed with ERCP to relieve the obstruction by placing a plastic biliary stent and performing brushing cytology to confirm the nature of the mass.

The patient was started on intravenous (IV) hydration and broad-spectrum antibiotics (meropenem) in preparation for the procedure. ERCP was successfully performed the following day, achieving biliary decompression through the placement of a plastic stent. Brush cytology was also taken during the ERCP for further evaluation of the biliary stricture. Within 24 hours post-ERCP, the patient developed generalized weakness, oliguria, and nausea. Repeat laboratory testing revealed significant metabolic disturbances, including acute kidney injury, hyperkalemia, hyperphosphatemia, hyperuricemia, and severe hypocalcemia, consistent with TLS (Table 2).

**Table 2:** Laboratory values 24 hours post-ERCP ERCP: endoscopic retrograde cholangiopancreatography; WBC: white blood cells, Alk phos: alkaline phosphatase, GGT: gamma-glutamyl transferase, CRP: C-reactive protein

Parameter	Value	Normal range
WBC	15200 cells/µL	4,000-11,000 cells/µL
Creatinine	2.3 mg/dL	0.6-1.2 mg/dL
Potassium	6.5 mmol/L	3.5-5.0 mmol/L
Phosphate	5.2 mg/dL	2.5-4.5 mg/dL
Uric Acid	8.1 mg/dL	3.5-7.2 mg/dL
Calcium	6.2 mg/dL	8.5-10.2 mg/dL
Direct bilirubin	8.9 mg/dL	< 0.3 mg/dL
Alk phos	382 U/L	45-115 U/L
GGT	265 U/L	9-48 U/L
CRP	137 mg/L	< 10 mg/L

Despite prompt recognition and aggressive management, including hydration, electrolyte correction, and renal support, the patient's condition continued to deteriorate rapidly. Rasburicase was not used because it was not available in Lebanon at the time. Nevertheless, the patient received aggressive hydration, electrolyte correction, and renal support, which are the mainstays of TLS management. Dialysis was considered; however, given the rapid deterioration and poor prognosis, the decision was made to focus on supportive care. Unfortunately, the patient passed away 24 hours later. The patient's rapid clinical deterioration was consistent with TLS, leading to multi-organ failure.

One week later, the brush cytology confirmed the diagnosis of cholangiocarcinoma.

This case illustrates the development of TLS in the setting of ERCP for cholangiocarcinoma, a rare but critical complication requiring immediate recognition and management.

## Discussion

TLS is a well-documented oncologic emergency that typically occurs in hematologic malignancies following chemotherapy but is rarely associated with solid tumors such as cholangiocarcinoma [7]. This case presents an unusual occurrence of TLS triggered by ERCP, a procedure commonly performed for biliary decompression. TLS, in this context, is believed to result from the physical manipulation of the tumor and the release of intracellular components, including potassium, phosphate, and uric acid, into the bloodstream [8]. This metabolic surge overwhelms the kidneys’ ability to excrete these substances, leading to acute kidney injury (AKI), hyperkalemia, hyperphosphatemia, hyperuricemia, and secondary hypocalcemia caused by phosphate precipitation with calcium [9].

Initially, sepsis- or cholangitis-induced AKI was considered. The patient presented with low-grade fever and cholangitis but was hemodynamically stable, ruling out septic shock. The nephrologist identified TLS as the primary cause due to several key factors. Firstly, the rapid onset of electrolyte disturbances was indicative of TLS, contrasting with the more gradual progression typically seen in AKI. Secondly, urine analysis revealed uric acid crystals, which are characteristic of TLS, rather than the granular casts or proteinuria often associated with AKI. Additionally, there were no clinical signs of severe infection such as high fever or hemodynamic instability, which are typically present in cases of sepsis-induced AKI. Blood cultures and infection markers returned negative, and the procalcitonin level was low at 0.2 ng/mL (normal range: <0.1 ng/mL), further ruling out sepsis as a primary cause.

The patient’s condition highlights a significant but underrecognized risk in individuals with a high tumor burden undergoing invasive procedures. According to Alqurashi et al. [7], TLS has been reported to occur spontaneously in 24% of solid tumor cases, though the majority of cases involve hematologic malignancies. This finding underscores the need for heightened vigilance in patients with bulky or metabolically active tumors. In our case, the diagnosis of TLS was confirmed based on the Cairo-Bishop criteria, which emphasize a combination of laboratory and clinical findings indicative of TLS, such as significant elevations in uric acid, potassium, and phosphate levels, along with acute renal failure [6].

Management of TLS relies on early recognition and intervention to prevent irreversible complications. Initial treatment involves aggressive intravenous hydration to enhance renal clearance of metabolic byproducts, correction of electrolyte imbalances, and administration of urate-lowering agents such as rasburicase or allopurinol to manage hyperuricemia. In severe cases, particularly those involving refractory hyperkalemia or persistent metabolic derangements, renal replacement therapy may be required [10]. This case underscores the importance of preemptive measures, such as close monitoring of laboratory parameters before and after invasive procedures in high-risk patients, to ensure timely intervention.

The findings in this patient demonstrate that TLS, though rare in solid tumors, should be included in the differential diagnosis for AKI and metabolic disturbances following ERCP in patients with significant tumor burden. Increased awareness among clinicians and early multidisciplinary involvement is critical to optimizing outcomes and reducing morbidity and mortality associated with this potentially fatal syndrome.

## Conclusions

This case underscores the importance of recognizing TLS as a rare but potentially fatal complication in patients with solid tumors, particularly following invasive procedures such as ERCP. While TLS is commonly associated with hematologic malignancies and chemotherapy, it can also occur in solid tumors due to mechanical tumor disruption and rapid cellular turnover. Early diagnosis and prompt management, including hydration, correction of electrolyte imbalances, and the use of urate-lowering therapies, are crucial to prevent severe complications, such as renal failure, cardiac arrhythmias, and metabolic crises.

This case serves as a reminder for clinicians to maintain a high index of suspicion for TLS in patients with significant tumor burden undergoing therapeutic interventions. Ensuring close monitoring and multidisciplinary collaboration is essential to optimizing patient outcomes. By recognizing this uncommon manifestation, clinicians can enhance the safety and efficacy of interventions in complex oncologic patients.
